# Glass: Home of the Periodic Table

**DOI:** 10.3389/fchem.2020.00384

**Published:** 2020-04-30

**Authors:** Georgiy Shakhgildyan, Alexey Lipatiev, Sergey Lotarev, Sergey Fedotov, Vladimir Sigaev

**Affiliations:** Department of Glass and Glass-Ceramics, Mendeleev University, Moscow, Russia

**Keywords:** Periodic Table, glass, glass-ceramics, glass formation, direct laser writing

## Abstract

Glass is the most common material around us, and humankind uses it every day for more than 5000 years. However, from the chemical point of view, glass is the only material that could represent almost all elements of the Periodic Table inside itself, showing the effect of the Periodic Law on properties of the final material. In this paper, we show the most remarkable examples demonstrating that glass can rightfully be called “home” for all chemical elements providing different properties depending on its composition. We gave a new look at the Periodic Table and described how a small number of glass-forming components creates unique glass structure which could enclose almost all remaining elements including transition and noble metals, lanthanides and actinides as modifying components providing an inconceivable number of discoveries in material science. Moreover, we reviewed a series of studies on the direct femtosecond laser writing in glasses which paves the way for a redistribution of chemical elements in the spatially confined nanosized zone in glass volume providing unique properties of laser-induced structures. Finally, for the first time, we reproduce the Periodic Table in birefringence colors in the bulk of silica glass using a direct laser writing technique. This image of 3.6 × 2.4 mm size can withstand temperature up to 900°C, humidity, electromagnetic fields, powerful cosmic and reactor radiation and other environmental factors and demonstrates both the art of direct laser writing and symbolic role of glass as the safest and eternal home for the Periodic Table.

## Introduction

Glass has been known to mankind for more than 5000 years, and it is one of the most common materials in modern life. Despite its long history, glass remains one of the most interesting objects to study in the field of inorganic materials science. Thanks to the work of researchers around the world and the variety of chemical elements collected in the Periodic Table, new glass compositions and processing methods are produced every year, creating new materials for the development of vital and sustainable technologies. According to theoretical calculations, the number of possible compositions of glasses is so large that the synthesis of all compositions would require the number of atoms close to the maximum theoretical content of atoms in the whole Universe (Zanotto and Coutinho, 2004). Understanding the importance of glass science and technology, D. I. Mendeleev himself was actively engaged in the development of new glass-based materials, his main results were reflected in his 1864s book “Glass Production” (Mendeleev, 1864).

In this paper, in honor of the recent anniversary of the Periodic Table of chemical elements, we briefly describe how a large class of glasses can be classified into groups of chemical elements of the Periodic Table and give a look on the perspective developments in the field of glass which make it one of the most promising materials of our time.

## Glass Families and Their Place in the Periodic Table

As mentioned above, almost all elements of the Periodic Table can be used for the production of glass, which makes it a kind of “home” for all elements. Depending on the type of chemical compounds that form glasses, they are divided into classes, each of which has a unique structure and properties that determine their application.

The most common class of glasses is oxide glasses. They are formed by structural units that are compounds of various elements with oxygen. These elements primarily include Si, Ge, B, P, As, their oxides are easily obtained in a glassy state and most glasses are produced on their basis. Also, oxides of Te, Ti, Se, Sb, Mo, W, Bi, Al, Ga, V, and other elements can act as glass-forming agents, either individually or in a mixture with other oxides. Depending on the type of the main glass-forming oxide, the name of the group of glasses is derived—silicate, phosphate, tellurite, etc.

A special place among oxide glasses is occupied by fused silica or silica glass (SiO_2_). It is the most refractory glass, it has a wide transparency window in the optical region and an abnormally low coefficient of thermal expansion (CTE) equal to 5.5·10^−7^/K^−1^(LeLosq et al., 2019). These determine its wide range of applications—from UV lamps to optical elements. It is thanks to the development of silica glass technology and the production of optical fibers that the worldwide implementation of broadband Internet access has become possible (Ballato and Dragic, 2016).

Silicate glasses which include oxides of various chemical elements from groups 1 to 17 are the most extensive group of glasses. In addition to Si and O, the most important elements of silicate glasses are alkaline (Li, Na, K) and alkaline earth (Mg, Ca, Sr, Ba) metals, as well as elements Al and B. Due to high transparency, acceptable strength and relatively low cost of production, products made of silicate glasses are present in all areas of human life: construction and transport, storage of products, machinery and much more. Ion exchange technology which includes replacement of small metal ions with larger ones in the surface layer of silicate glasses allows to significantly increase glass strength, which is used to create flexible mobile devices (Mauro and Morten, 2016). Using silicate glasses and different thin-film layers smart glasses are developed. Smart glass technology enables the fabrication of glass-based devices to control passing light. This can considerably decrease the building's energy demands with improving the indoor environment (Rezaei et al., 2017). The introduction of various chemical elements into the glass composition (mainly transition, rare earth, and noble metals), leads to the coloring of glasses. Even elements of the actinide group can be used for the production of glasses. Thus, U_2_O_3_ has long been used for the production of tableware, jewelry and optical filters, since U ions give a bright green color to the glass (Strahan, 2001). Currently, the ability of silicate glasses to contain radioactive elements (U, Pu) is used for the disposal of radioactive waste in vitrification technology (Gin et al., 2013). If it is necessary, some elements can be even converted to a radioactive state in glasses, thereby creating radiopharmaceuticals. Thus, glass microspheres with the Y^90^ isotope for liver cancer treatment are produced using Y_2_O_3_-Al_2_O_3_-SiO_2_ glass (Ehrhardt and Day, 1987; Sigaev et al., 2012). Moreover, utilizing silicate glasses containing oxides of Al, Li, Mg, Ca, Ti, Zr, P, and other elements, glass-ceramics can be made by the bulk nucleated crystallization routine. Glass-ceramics demonstrate different combinations of properties [high strength, zero porosity, precision-regulated coefficient of thermal expansion (CTE), including near-zero values, biocompatibility, etc.] and find applications in various fields from medicine to astronomy (Zanotto, 2010).

The industrial production of non-silica oxide glasses is less widespread. Meanwhile, new challenges in science and technology lead to the development of such glasses. TeO_2_-based glasses have an increased refractive index (>2.0) and are used in complex optical devices in the visible and near-IR range. GeO_2_-based glasses are actively being studied to create new optical and laser components (Sigaev et al., 2011; Starobor et al., 2016). Borate glasses (based on B_2_O_3_) with additives of different oxides are used in medicine for the restoration of soft tissues, in optics and photonics (Lorenzi et al., 2018; Feller, 2019). Phosphate glasses (based on P_2_O_5_) containing oxides of rare earth elements (Nd, Eu, Tb, Er, Yb) are used in industry for the production of active elements of high-power lasers (Campbell and Suratwala, 2000). Phosphate and borate glasses with additives of Zn, Sn, B, Al, Na, K oxides are widely used for: sealing materials with a variety of CTE values, biomedical applications and the development of solid electrolytes for the new generation of batteries (Muñoz et al., 2019).

Glasses formed by the elements of group 16 (S, Se, Te) together with elements of group 14 and 15 (Ge, Sn, P, As, Sb) belong to the class of chalcogenide glasses. These glasses have ultrahigh transparency in the IR range (up to 25 microns), which determines their use as IR imaging devices in new generation thermal imagers, optical sensors for IR spectroscopy and active elements of fiber CO_2_ lasers (Hubert et al., 2011).

The class of halide glasses includes materials based on elements of group 17 (primarily F, also Cl, Br, I) in the combination with metals (Be, Zr, Hf, Ba, La, Al, Y). The most widely studied glass composition is ZBLAN (in the ZrF_4_-BaF_2_-LaF_3_-AlF_3_-NaF system). Production of such glasses is complicated by their high tendency to crystallization and extremely high requirements for the degree of purity of raw materials. However, research in this area is justified by the possibility of obtaining optical fibers with minimal optical losses which could provide a significant (up to 100 times) increase in data transfer speed compared to current fiber cables and open the way for a new generation of near-IR fiber lasers (Wetenkamp et al., 1992; Clare et al., 2019). It is worth noting that the researchers have established the possibility of obtaining a defect-free fiber from ZBLAN glass in the absence of gravity and are currently conducting experiments to obtain such a fiber on the International space station (Starodubov et al., 2014).

Metal glasses are the general name of a wide class of amorphous materials consisting of metals or alloys of metals and metalloids, including elements of groups 2–15 (the most common are Zr, Ti, Cu, Ni, Be, Mg, Fe, Co, etc.). Metal glasses have high crystallization tendency which could be overcome by unusually high melt cooling rates (from 10^5^ to 10^12^ °C/s). The advantages of metal glasses over metals and metal alloys are high values of mechanical strength, corrosion resistance, magnetic properties and electrical resistance. Currently, it is possible to produce not only metal glasses in the form of thin plates but bulk products with complex surface morphology (Greer, 1995; Schroers, 2013).

Thus, almost all elements of the Periodic Table in various ratios and compounds can be part of the glass, changing its structure and giving unique properties to the material. The abundance of variations in the composition and methods of glass production opens up new directions for its use in the promising fields of optics, medicine, photonics, energy and many others.

## Direct Laser Writing in Glass and Redistribution of Elements

In addition to changing the chemical composition of glass, post-processing methods are important technologies for creating materials with specified properties. Using methods of ion exchange and surface coatings, it is possible to create high-strength and energy-efficient materials with specified optical properties. At once, the possibility of micro- and even nanoscale control of glass properties opens up previously impossible ways for the development of new materials and devices. Due to the intensive evolution of femtosecond laser technology, a wide field of research has emerged into the interaction of ultrashort laser pulses with glasses. Through a multiphoton mechanism, the energy of focused ultrashort laser pulses is absorbed by glass in the focus area. In this area, laser intensity exceeds the values of 10^13^ W/cm^2^, resulting in modification of the glass structure. The non-linear nature of the absorption of ultrashort pulses makes it possible to control the spatial position of modified regions in the volume of glass, which opens up prospects for their three-dimensional (3D) modification at the micro- and nanoscale by so-called direct laser writing (DLW) technique (Osellame et al., 2012; Sugioka and Cheng, 2014; Phillips et al., 2015).

In recent years, studies on DLW of various structures in glasses have revealed a wide range of phenomena that occur when the material absorbs ultrashort pulses. These include local changes in the refractive index and writing of optical waveguides (Phillips et al., 2015), the formation of surface and bulk birefringent nanoperiodic structures (nanogratings) (Shimotsuma et al., 2003; Lotarev et al., 2019a), formation of micro-and nanobubbles (Bellouard and Hongler, 2011), local crystallization of non-linear phases in glass (Lipatiev et al., 2018b, 2020; Lotarev et al., 2019b), precipitation of metal clusters, nanoparticles, and semiconductor quantum dots (Marquestaut et al., 2014; Shakhgildyan et al., 2018; Vetchinnikov et al., 2018; Hu et al., 2019). Despite the physical nature of the processes, involved in the interaction of laser pulses with glass, they lead to a local change in the chemical composition of glass by ions migrations to different areas of the laser affected zone. According to the latest studies, glass-modifier ions (Na, K, Mg, Ca, Sr, Ba, Zr) are most susceptible to migration. When observed in a plane perpendicular to the laser beam, such ions migrate to the periphery of the laser affected zone, while glass-forming ions tend to concentrate in the center (Fernandez et al., 2018; Lotarev et al., 2018). The most promising application of DLW in glasses is multidimensional optical data storage with a high capacity and almost unlimited lifetime. This method is based on the periodic nanostructures (nanogratings) laser-written in silica glass (Shimotsuma et al., 2003). Nanogratings are formed by periodic nanoporous regions interlaced with solid glass. They are oriented perpendicular to the plane of polarization of the writing beam and exhibiting form birefringence which can be used for the data encoding (Zhang et al., 2014). In order to increase data writing speed, it was proposed to use nanoporous SiO_2_ glass as storage media instead of silica glass (Fedotov et al., 2018; Lipatiev et al., 2018a). In this case, birefringent sub-micron hollow cavities were formed under the action of ultrafast laser pulses. The above-mentioned promising results served as the basis for launching applied research projects aimed at creating glass-based optical memory technology. The first project of this kind was launched in the Mendeleev University in Russia (Project “Quartz”) Lotarev (2017), next in the Microsoft laboratories (Project “Silica”) Project Silica (2017).

Thus, using various combinations of chemical elements for glass production and laser processing methods, it is possible to create new materials and devices for a wide range of applications. First of all, they include secure data storage, as well as elements of integrated photonic circuits (waveguides, interferometers, switches), optical sensors, microfluidic devices, and much more. The processes occurring during the interaction of femtosecond laser radiation with glass, in most cases, lead to a local change in its chemical composition and migration of chemical elements to certain zones limited by the laser spot, which largely determines the changed properties of the resulting region. At the same time, the ability of a fine-tuning of glass composition at the production stage allows to ensure the most efficient flow of DLW processes.

## Eternal and Miniature Periodic Table

To symbolically mark the possibility of using almost all elements of the Periodic Table in glass, we inscribed a color image in polarized light of the Periodic Table in the volume of commercially available KU-1 type silica glass (TechnoQuartz Ltd., Russia) using the DLW method. This writing technology is based on the birefringent properties of laser-induced nanogratings and stress, due to which it is possible to vary the wavelength of light passing through the polarizer and light analyzer (Spring et al., 2010; Sun et al., 2020).

In this work, the Periodic Table was written in a 10 mm thick silica glass sample at a depth of 900 microns from the surface by a laser beam focused with a Mitutoyo MPlan APO 5X lens (N. A. = 0.14). We used Pharos 6 W femtosecond laser (Light Conversion Dev.) with a 1,030 nm central wavelength. To control the color of cells via their birefringence in the Periodic Table, the energy of laser pulses, the number of layers, and the distance between them were varied. The scanning speed of the laser beam was 2 mm/s. The writing conditions of certain groups of elements are shown in Table 1. It should be mentioned that Table 1 represents the actual interlayer shift whereas the corresponding shift of the sample during writing of the Periodic Table was equal to these values divided by 1.45 taking the refraction into account because the refractive index of silica glass is 1.45 at 1030 nm wavelength. The names of the elements were written using tighter focusing of the laser beam by means of an Olympus LCPLNIR 20X lens (N. A. = 0.45) and with the relative rotation of the laser beam polarization plane by 45°.

**Table 1:** Writing conditions for the DLW of the Periodic Table in the sample of silica glass.

**Group of elements**	**Number of layers**	**Shift between layers, μm**	**Pulse energy, μJ**
Alkali and alkali earth metals	1	–	4.8
Transition metals	2	100	4.2
Non-metals	3	130	4.2
Lanthanides and Actinides	2	175	4.2
Post-transition metals	2	290	4.2
Noble gases	2	44	4.2

**Figure 1:**
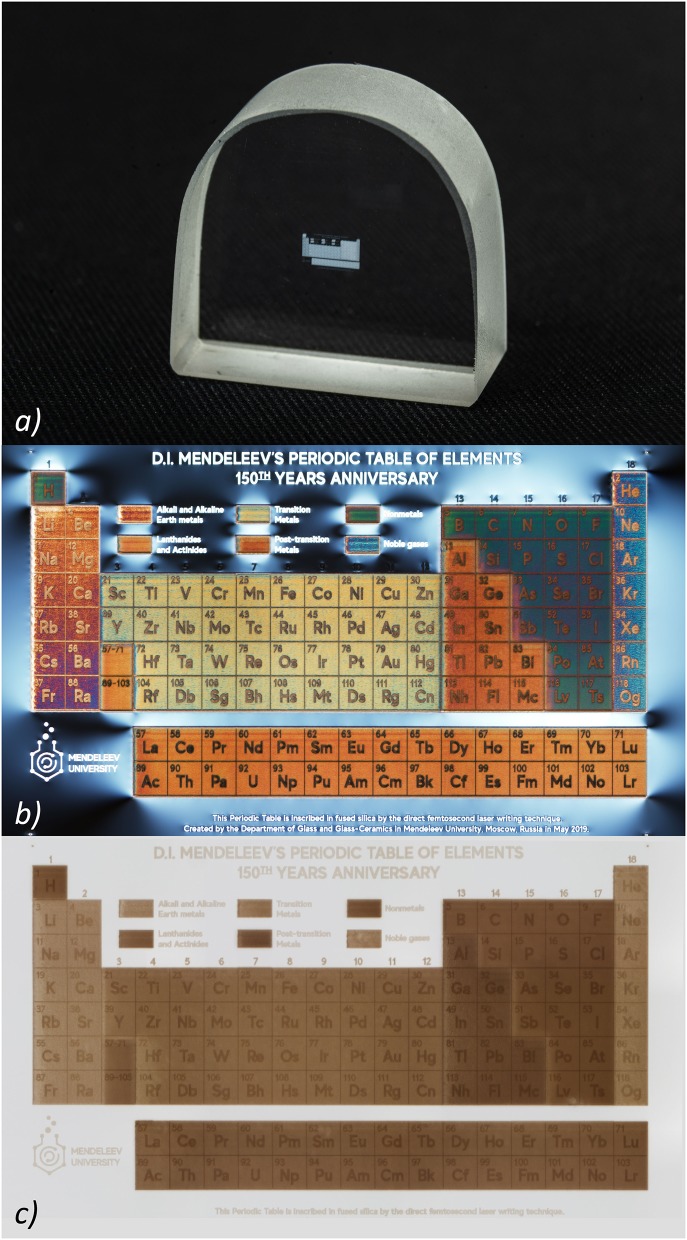
**(a)** Real-size image of the silica glass sample with the laser written Periodic Table; **(b,c)** Optical microscopy image of the Periodic Table laser written in the silica glass sample captured with and without crossed polarizers, respectively. The length of the one cell is 200 μm.

The photo of the glass sample with the written Table is shown in Figure 1a. The total size of the Table is 3.6 × 2.4 mm. To see the Table in color, it is necessary to observe it through a microscope with crossed polarizers (Figure 1b), while the image of the Table without crossed polarizers is shown in Figure 1c. The size of each Table cell is 200 × 200 microns. Annealing of a glass sample with a written Table at 900°C for 1 h does not lead to noticeable degradation of the image nevertheless its color was slightly changed due to the relaxation of laser-induced stress. After repeated thermal shocks (rapid cooling from 900°C to cold water) of the glass sample with the written Table completely retains its integrity. Due to the high chemical and radiation stability of silica glass, written image is also not subjected to degradation under the influence of moisture, acids, alkalis and radiation exposure (within the stability of silica glass). Thus, on the one hand, glass can be produced using almost all elements of the Periodic Table, and on the other, thanks to the DLW method, glass becomes the eternal storage of the Periodic Table.

## Discussion and Conclusion

Despite the long history, today glass is the most important class of materials that is in demand in various industries. The variety of properties and applications of glass is associated with an unimaginably large number of possible compositions—combinations of various chemical elements. New opportunities for creating materials based on glass with unique properties are introduced by laser modification methods, which allow to create various multifunctional structures and objects in the glass volume.

In honor of the 150th anniversary of the Periodic Table of chemical elements, we showed the possibility of creating a color image of the Periodic Table in silica glass using direct laser writing technology. This image covers an area of 3.6 × 2.4 mm, while the size of each Table cell is 200 × 200 microns. Annealing of a glass sample with a written Table at 900°C for 1 h does not lead to noticeable degradation of the image. After repeated thermal shocks of the glass sample with the Table it completely retains its integrity. Due to the high chemical and radiation stability of silica glass, the written image is also not subjected to degradation under the influence of moisture, acids, alkalis and radiation exposure (within the stability of silica glass). Therefore, the created Table image can be considered eternal.

Thus, we want to show that on the one hand, glass can be produced using almost all elements of the Periodic Table, and on the other, that thanks to the DLW method, glass becomes the eternal storage of the Periodic Table. With these conclusions, we want to emphasize the importance of developing research in the field of glassy materials, which, thanks to the variety of elements of the Periodic Table and the use of new technologies, ensure the ever-accelerating development of the material science, instrumentation and information base of mankind.

## Data Availability Statement

The datasets generated for this study are available on request to the corresponding author.

## Author Contributions

GS, AL, SL, and VS contributed the conception and design of the study. Experimental work was carried out by AL and SF (programming, modeling, and laser writing) under supervision of SL and VS. GS wrote the manuscript and prepared images with contributions of AL, SL, and VS in certain sections. All authors participated in the analysis and discussion of obtained results.

## Conflict of Interest

The authors declare that the research was conducted in the absence of any commercial or financial relationships that could be construed as a potential conflict of interest.

## References

[B1] Ballato J. Dragic P. (2016). Glass: the carrier of light - a brief history of optical fiber. Int. J. Appl. Glass Sci. 7, 413–422. 10.1111/ijag.12239

[B2] Bellouard Y. Hongler M.-O. (2011). Femtosecond-laser generation of self-organized bubble patterns in fused silica. Opt. Express 19, 6807–6821. 10.1364/oe.19.006807 21451708

[B3] Campbell J. H. Suratwala T. I. (2000). Nd-doped phosphate glasses for high-energy/high-peak-power lasers. J. Non Cryst. Solids 263–264, 318–341. 10.1016/S0022-3093(99)00645-6

[B4] Clare A. G. Wachtel P. F. Musgraves J. D. (2019). “Halide glasses,” in Springer Handbook of Glass, eds Musgraves J. D. Hu J. Calvez L. (Springer International Publishing), 595–616. 10.1007/978-3-319-93728-1_17.

[B5] Ehrhardt G. J. Day D. E. (1987). Therapeutic use of ^90^Y microspheres. Int. J. Rad. Appl. Instrum. B 14, 233–242. 10.1016/0883-2897(87)90047-x 3667306

[B6] Fedotov S. S. Okhrimchuk A. G. Lipatiev A. S. Stepko A. A. Piyanzina K. I. Shakhgildyan G. Y.. (2018). 3-bit writing of information in nanoporous glass by a single sub-microsecond burst of femtosecond pulses. Opt. Lett. 43:851. 10.1364/ol.43.000851 29444010

[B7] Feller S. (2019). “Borate glasses,” in Springer Handbook of Glass, eds Musgraves J. D. Hu J. Calvez L. 505–524. 10.1007/978-3-319-93728-1_14

[B8] Fernandez T. T. Sakakura M. Eaton S. M. Sotillo B. Siegel J. Solis J. (2018). Bespoke photonic devices using ultrafast laser driven ion migration in glasses. Progr. Mater. Sci. 94, 68–113. 10.1016/j.pmatsci.2017.12.002

[B9] Gin S. Abdelouas A. Criscenti L. J. Ebert W. L. Ferrand K. Geisler T. (2013). An international initiative on long-term behavior of high-level nuclear waste glass. Mater. Today 16, 243–248. 10.1016/j.mattod.2013.06.008

[B10] Greer A. L. (1995). Metallic glasses. Science 267, 1947–1953. 10.1126/science.267.5206.1947 17770105

[B11] Hu Y. Zhang W. Ye Y. Zhao Z. Liu C. (2019). Femtosecond-laser-induced precipitation of CsPbBr3 perovskite nanocrystals in glasses for solar spectral conversion. ACS Appl. Nano Mater. 3, 850–857. 10.1021/acsanm.9b02362

[B12] Hubert M. Delaizir G. Monnier J. Godart C. Ma H. L. Zhang X. H.. (2011). An innovative approach to develop highly performant chalcogenide glasses and glass-ceramics transparent in the infrared range. Opticsexpress 19, 23513–23522. 10.1364/OE.19.023513 22109229

[B13] LeLosq C. Cicconi M. R. Greaves G. N. Neuville D. R. (2019). “Silicate glasses,” in Springer Handbook of Glass, eds Musgraves J. D. Hu J. Calvez L. 441–503. 10.1007/978-3-319-93728-1_13

[B14] Lipatiev A. S. Fedotov S. S. Okhrimchuk A. G. Lotarev S. V. Vasetsky A. M. Stepko A. A.. (2018a). Multilevel data writing in nanoporous glass by a few femtosecond laser pulses. Appl. Opt. 57, 978–982. 10.1364/ao.57.000978 29400776

[B15] Lipatiev A. S. Lotarev S. V. Smayev M. P. Lipateva T. O. Karateev I. A. Presnyakov M. Y. (2020). Space-selective crystallization of glass by an optical vortex beam. CrystEngComm 22, 430–434. 10.1039/c9ce01869g

[B16] Lipatiev A. S. Moiseev I. A. Lotarev S. V. Lipateva T. O. Presnyakov M. Y. Fedotov S. S. (2018b). Growth of fresnoite single crystal tracks inside glass using femtosecond laser beam followed by heat treatment. Cryst. Growth Des. 18, 7183–7190. 10.1021/acs.cgd.8b01358

[B17] Lorenzi R. Golubev N. V. Ziaytdinova M. Z. Jar,ý V. Babin V. Malashkevich G. E. (2018). Radio- and photoluminescence properties of Ce/Tb co-doped glasses with huntite-like composition. Opt. Mater. 78, 247–252. 10.1016/j.optmat.2018.02.016

[B18] Lotarev S. (2017). In Search of Permanent Memory: From Cuneiform on Clay to Nanostructures in Glass. Available online at: https://www.kommersant.uk/articles/in-search-of-permanent-memory-from-cuneiform-on-clay-to-nanostructures-in-glass (accessed March 15, 2020).

[B19] Lotarev S. Fedotov S. Lipatiev A. Presnyakov M. Kazansky P. Sigaev V. (2018). Light-driven nanoperiodical modulation of alkaline cation distribution inside sodium silicate glass. J. Non Cryst. Solids 479, 49–54. 10.1016/j.jnoncrysol.2017.10.008

[B20] Lotarev S. V. Fedotov S. S. Kurina A. I. Lipatiev A. S. Sigaev V. N. (2019a). Ultrafast laser-induced nanogratings in sodium germanate glasses. Opt. Lett. 44, 1564–1567. 10.1364/ol.44.001564 30933091

[B21] Lotarev S. V. Lipatiev A. S. Lipateva T. O. Fedotov S. S. Naumov A. S. Moiseev I. A. (2019b). Ultrafast-laser vitrification of laser-written crystalline tracks in oxide glasses. J. Non Cryst. Solids 516, 1–8. 10.1016/j.jnoncrysol.2019.04.027

[B22] Marquestaut N. Petit Y. Royon A. Mounaix P. Cardinal T. Canioni L. (2014). Three-dimensional silver nanoparticle formation using femtosecond laser irradiation in phosphate glasses: analogy with photography. Adv. Funct. Mater. 24, 5824–5832. 10.1002/adfm.201401103

[B23] Mauro J. C. Morten M. S. (2014). Ultra-Thin Strengthened Glasses. U.S. Patent Application No. 13/961, 211.

[B24] Mendeleev D. I. (1864). Glass Production. Saint Petersburg: Public benefit.

[B25] Muñoz F. Rocherullé J. Ahmed I. Hu L. (2019). “Phosphate glasses,” in Springer Handbook of Glass, eds Musgraves J. D. Hu J. Calvez L. 553–594. 10.1007/978-3-319-93728-1_16

[B26] Osellame R. Cerullo G. Ramponi R. (Eds.). (2012). “Femtosecond laser micromachining,” in Topics in Applied Physics (Berlin; Heidelberg: Springer). 10.1007/978-3-642-23366-1

[B27] Phillips K. C. Gandhi H. H. Mazur E. Sundaram S. K. (2015). Ultrafast laser processing of materials: a review. Adv. Opt. Photonics 7:684 10.1364/aop.7.000684

[B28] Project Silica (2017). Microsoft Research. Available online at: https://www.microsoft.com/en-us/research/project/project-silica/c (accessed March 15, 2020).

[B29] Rezaei S. D. Shannigrahi S. Ramakrishna S. (2017). A review of conventional, advanced, and smart glazing technologies and materials for improving indoor environment. Solar Energy Mater. Solar Cells 159, 26–51. 10.1016/j.solmat.2016.08.026

[B30] Schroers J. (2013). Bulk metallic glasses. Phys. Today 66, 32–37. 10.1063/pt.3.1885

[B31] Shakhgildyan G. Y. Lipatiev A. S. Vetchinnikov M. P. Popova V. V. Lotarev S. V. Golubev N. V. (2018). One-step micro-modification of optical properties in silver-doped zinc phosphate glasses by femtosecond direct laser writing. J. Non Cryst. Solids 481, 634–642. 10.1016/j.jnoncrysol.2017.12.011

[B32] Shimotsuma Y. Kazansky P. G. Qiu J. Hirao K. (2003). Self-organized nanogratings in glass irradiated by ultrashort light pulses. Phys. Rev. Lett. 91:247405. 10.1103/physrevlett.91.247405 14683157

[B33] Sigaev V. N. Atroschenko G. N. Savinkov V. I. Sarkisova P. D. Babajewb G. Lingel K. (2012). Structural rearrangement at the yttrium-depleted surface of HCl-processed yttrium aluminosilicate glass for ^90^Y-microsphere brachytherapy. Mater. Chem. Phys. 133, 24–28. 10.1016/j.matchemphys.2011.12.079

[B34] Sigaev V. N. Golubev N. V. Ignat'eva E. S. Savinkov V. I. Campione M. Lorenzi R.. (2011). Nickel-assisted growth and selective doping of spinel-like gallium oxide nanocrystals in germano-silicate glasses for infrared broadband light emission. Nanotechnology 23:15708. 10.1088/0957-4484/23/1/015708 22155977

[B35] Spring K. R. Parry-Hill M. J. Davidson M. W. (2010). Michel-Levy Birefringence Chart. Olympus Microscopy Resource Center. Available online at: http://olympus.magnet.fsu.edu/primer/java/polarizedlight/michellevy/index.html

[B36] Starobor A. V. Zheleznov D. S. Palashov O. V. Savinkov V. I. Sigaev V. N. (2016). Borogermanate glasses for Faraday isolators at high average power. Opt. Commun. 358, 176–179. 10.1016/j.optcom.2015.09.047

[B37] Starodubov D. Mechery S. Miller D. Ulmer C. Willems P. Ganley J. (2014). ZBLAN fibers: from zero gravity tests to orbital manufacturing. Appl. Ind. Optics. 10.1364/aio.2014.am4a.2

[B38] Strahan D. (2001). Uranium in glass, glazes and enamels: history, identification and handling. Stud. Conserv. 46, 181–195. 10.1179/sic.2001.46.3.181

[B39] Sugioka K. Cheng Y. (2014). Ultrafast lasers—reliable tools for advanced materials processing. Light Sci. Appl. 3:e149 10.1038/lsa.2014.30

[B40] Sun Q. Lee T. Beresna M. Brambilla G. (2020). Control of laser induced cumulative stress for efficient processing of fused silica. Sci. Rep. 10:3819. 10.1038/s41598-020-60828-3 32123266 PMC7051964

[B41] Vetchinnikov M. P. Lipatiev A. S. Shakhgildyan G. Y. Golubev N. V. Ignat'eva E. S. Fedotov S. S.. (2018). Direct femtosecond laser-induced formation of CdS quantum dots inside silicate glass. Opt. Lett. 43, 2519–2522. 10.1364/ol.43.002519 29856419

[B42] Wetenkamp L. West G. F. Többen H. (1992). Optical properties of rare earth-doped ZBLAN glasses. J. Non Cryst. Solids 140, 35–40. 10.1016/s0022-3093(05)80737-9

[B43] Zanotto E. D. (2010). Bright future for glass-ceramics. Am. Ceramics Soc. Bull. 89, 19–27.

[B44] Zanotto E. D. Coutinho F. A. B. (2004). How many non-crystalline solids can be made from all the elements of the periodic table? J. Non Cryst. Solids 347, 285–288. 10.1016/j.jnoncrysol.2004.07.081

[B45] Zhang J. Gecevičius M. Beresna M. Kazansky P. G. (2014). Seemingly unlimited lifetime data storage in nanostructured glass. Phys. Rev. Lett. 112:033901. 10.1103/PhysRevLett.112.033901 24484138

